# *N*,*N*,*N*′-Trimethyl-*N*′′-(4-nitro­phen­yl)-*N*′-phenyl­guanidine

**DOI:** 10.1107/S160053681400693X

**Published:** 2014-04-05

**Authors:** Ioannis Tiritiris, Wolfgang Frey, Willi Kantlehner

**Affiliations:** a Fakultät Chemie/Organische Chemie, Hochschule Aalen, Beethovenstrasse 1, D-73430 Aalen, Germany; b Institut für Organische Chemie, Universität Stuttgart, Pfaffenwaldring 55, 70569 Stuttgart, Germany

## Abstract

The C—N bond lengths in the guanidine unit of the title compound, C_16_H_18_N_4_O_2_, are 1.298 (2), 1.353 (2) and 1.401 (3) Å, indicating double- and single-bond character. The N—C—N angles are 115.81 (16), 118.90 (18) and 125.16 (18)°, showing a deviation of the CN_3_ plane from an ideal trigonal–planar geometry. In the crystal, C—H⋯O hydrogen bonds are observed between the methyl- and aromatic-H atoms and nitro-O atoms. One H atom of the phenyl ring and of the NMe_2_ group associate with the O atoms of the nitro group, giving chains along the *a-* and *b-*axis directions. Cross-linking of these two chains results in a two-dimensional network along *bc*.

## Related literature   

For the synthesis and characterization of compounds for blue OLEDs, see: Agarwal *et al.* (2011 ▶). For the crystal structures of *N*-methyl­ated di­phenyl­guanidines, see: Tanatani *et al.* (1998 ▶). For non-classical hydrogen bonds, see: Desiraju & Steiner (1999 ▶). For the crystal structure of *N*′′-(4-carbazol-9-yl-phen­yl)-*N*,*N*′-diethyl-*N*,*N*′-di­phenyl­guanidine, see: Tiritiris & Kantlehner (2013 ▶), and of *N*′′-(4-meth­oxy­phen­yl)-*N*,*N*,*N*′-trimethyl-*N*′-phenyl­guanidine, see: Tiritiris *et al.* (2014 ▶).
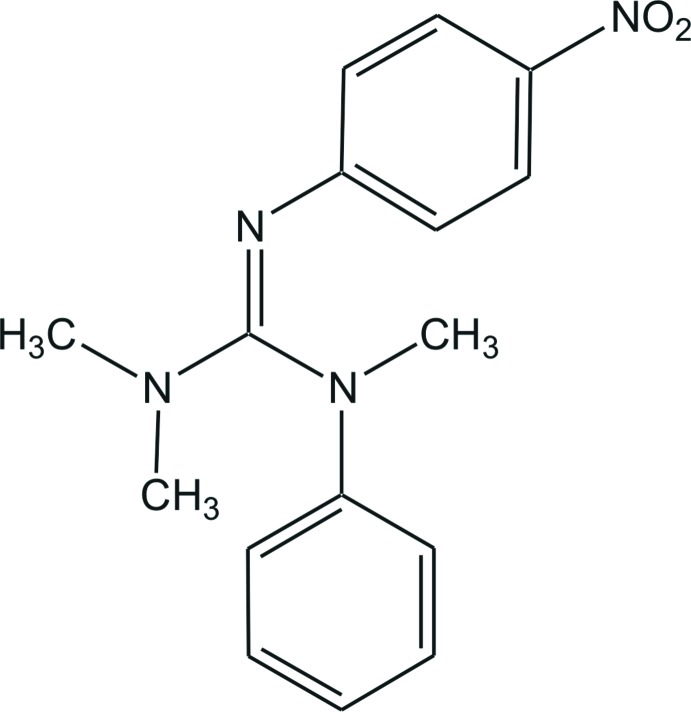


## Experimental   

### 

#### Crystal data  

C_16_H_18_N_4_O_2_
*M* *_r_* = 298.34
Monoclinic, 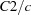

*a* = 18.409 (2) Å
*b* = 7.7140 (8) Å
*c* = 22.493 (3) Å
β = 109.503 (7)°
*V* = 3010.9 (6) Å^3^
*Z* = 8
Mo *K*α radiation
μ = 0.09 mm^−1^
*T* = 293 K
0.35 × 0.25 × 0.20 mm

#### Data collection  

Nicolet P3/F diffractometer
2974 measured reflections
2974 independent reflections
2237 reflections with *I* > 2σ(*I*)
3 standard reflections every 50 reflections intensity decay: 3%

#### Refinement  

*R*[*F* ^2^ > 2σ(*F* ^2^)] = 0.053
*wR*(*F* ^2^) = 0.124
*S* = 1.06
2974 reflections
203 parameters
H-atom parameters constrained
Δρ_max_ = 0.18 e Å^−3^
Δρ_min_ = −0.22 e Å^−3^

### 

Data collection: *XSCANS* (Siemens, 1996 ▶); cell refinement: *XSCANS*; data reduction: *SHELXTL* (Sheldrick, 2008 ▶); program(s) used to solve structure: *SHELXS97* (Sheldrick, 2008 ▶); program(s) used to refine structure: *SHELXL97* (Sheldrick, 2008 ▶); molecular graphics: *DIAMOND* (Brandenburg & Putz, 2005 ▶); software used to prepare material for publication: *SHELXL97*.

## Supplementary Material

Crystal structure: contains datablock(s) I, global. DOI: 10.1107/S160053681400693X/nr2049sup1.cif

Structure factors: contains datablock(s) I. DOI: 10.1107/S160053681400693X/nr2049Isup2.hkl

Supporting information file. DOI: 10.1107/S160053681400693X/nr2049Isup3.cml

CCDC reference: 994178

Additional supporting information:  crystallographic information; 3D view; checkCIF report

## Figures and Tables

**Table 1:** Hydrogen-bond geometry (Å, °)

*D*—H⋯*A*	*D*—H	H⋯*A*	*D*⋯*A*	*D*—H⋯*A*
C12—H12⋯O2^i^	0.93	2.49	3.416 (3)	173
C2—H2*A*⋯O1^ii^	0.96	2.72	3.064 (3)	102

Symmetry codes: (i) 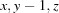; (ii) 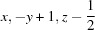.

**Fig. 1.**
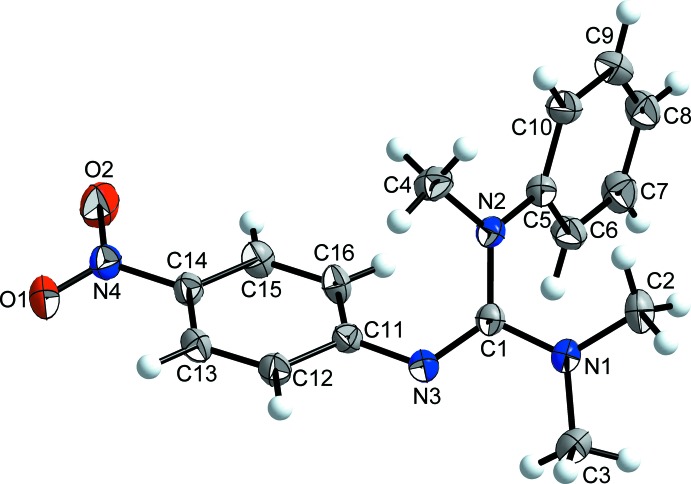
The structure of the title compound with atom labels and 50% probability displacement ellipsoids.

**Fig. 2.**
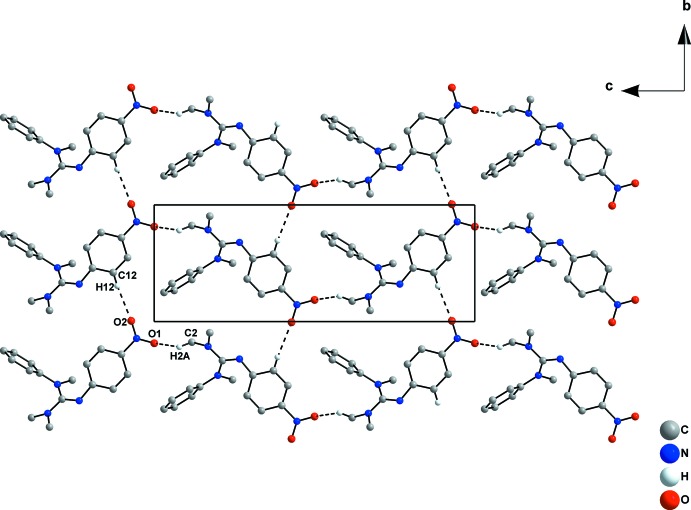
Packing diagram of the title compound. The C–H···O hydrogen bonds (indicated by dashed lines) are arranged in a two-dimensional network (bc-view). Only hydrogen atoms involved in the hydrogen bonding system are shown.

## supplementary crystallographic information

### 1. Comment

We were interested in the synthesis and characterization of aromatic guanidines to examine their suitability in OLEDs (Agarwal *et al.*, 2011). Because the crystal structure of the title compound was not known so far, it was decided to carry out an appropriate investigation. According to the structure analysis, the C1–N3 bond in the guanidine unit is 1.298 (2) Å, indicating double bond character. The bond lengths C1–N2 = 1.401 (3) Å and C1–N1 = 1.353 (2) Å are elongated and characteristic for C–N imine single bonds. The N–C1–N angles are 115.81 (16)° (N1–C1–N2), 125.16 (18)° (N2–C1–N3) and 118.90 (18)° (N1–C1–N3), showing a deviation of the CN_3_ plane from an ideal trigonal planar geometry (Fig. 1). Similar bond lengths and angles of the guanidine CN_3_ part have been found by structure analysis for *N*''-(4-Carbazol-9-yl-phenyl)- *N*,*N*'-diethyl-*N*,*N*'-diphenyl-guanidine (Tiritiris & Kantlehner, 2013), several *N*-methylated diphenylguanidines (Tanatani *et al.*, 1998) and *N*''- (4-methoxyphenyl)-*N*,*N*,*N*'-trimethyl-*N*'- phenylguanidine (Tiritiris *et al.*, 2014). Non-classical C–H···O hydrogen bonds (Desiraju & Steiner, 1999) between methyl hydrogen atoms, aromatic hydrogen atoms and oxygen atoms of the nitro groups are present [*d*(H···O) = 2.49 and 2.72 Å] (Tab. 1). One hydrogen atom of the phenyl ring (H12) is associated with the oxygen atom (O2) of the nitro group, resulting in chains along the *b* axis. A second hydrogen atom of the NMe_2_ group (H2A) is connected with O1, giving chains along the *a* axis. By crosslinking of both chains, a two-dimensional network along *bc* results (Fig. 2).

### 2. Experimental

One equivalent of *N*,*N*-dimethyl-*N*',*N*'-methylphenyl- chloroformamidinium-chloride (synthesized from *N*,*N*-dimethyl- *N*',*N*'-methylphenylthiourea and phosgene) was reacted with one equivalent of 4-nitroaniline (Sigma-Aldrich) in acetonitrile, in the presence of one equivalent triethylamine, at 273 K. The obtained mixture consisting of the guanidinium chloride and triethylammonium chloride was reacted in the next step with an excess of an aqueous sodium hydroxide solution at 273 K. After extraction of the guanidine with diethyl ether from the water phase, the solvent was evaporated and the title compound was isolated in form of a colourless solid. Single crystals have been obtained by recrystallization from a saturated acetonitrile solution at room temperature.

### 3. Refinement

The hydrogen atoms of the methyl groups were allowed to rotate with a fixed angle around the C–N bond to best fit the experimental electron density, with *U*_iso_(H) set to 1.5*U*_eq_(C) and d(C—H) = 0.96 Å. H atoms for C_aromatic_ were positioned geometrically and refined using riding model, with C—H = 0.93 Å and *U*_iso_(H) = 1.2*U*_eq_(C).

### Crystal data

**Table:**

C_16_H_18_N_4_O_2_	*F*(000) = 1264
*M**_r_* = 298.34	*D*_x_ = 1.316 Mg m^−^^3^
Monoclinic, *C*2/*c*	Mo *K*α radiation, λ = 0.71073 Å
Hall symbol: -C 2yc	Cell parameters from 35 reflections
*a* = 18.409 (2) Å	θ = 14–17°
*b* = 7.7140 (8) Å	µ = 0.09 mm^−^^1^
*c* = 22.493 (3) Å	*T* = 293 K
β = 109.503 (7)°	Plate, colorless
*V* = 3010.9 (6) Å^3^	0.35 × 0.25 × 0.20 mm
*Z* = 8	

### Data collection

**Table:**

Nicolet P3/F diffractometer	*R*_int_ = 0.000
Radiation source: sealed tube	θ_max_ = 26.0°, θ_min_ = 1.9°
Graphite monochromator	*h* = −22→21
Wyckoff scan	*k* = 0→9
2974 measured reflections	*l* = 0→27
2974 independent reflections	3 standard reflections every 50 reflections
2237 reflections with *I* > 2σ(*I*)	intensity decay: 3%

### Refinement

**Table:**

Refinement on *F*^2^	Secondary atom site location: difference Fourier map
Least-squares matrix: full	Hydrogen site location: inferred from neighbouring sites
*R*[*F*^2^ > 2σ(*F*^2^)] = 0.053	H-atom parameters constrained
*wR*(*F*^2^) = 0.124	*w* = 1/[σ^2^(*F*_o_^2^) + (0.0442*P*)^2^ + 2.9581*P*] where *P* = (*F*_o_^2^ + 2*F*_c_^2^)/3
*S* = 1.06	(Δ/σ)_max_ < 0.001
2974 reflections	Δρ_max_ = 0.18 e Å^−^^3^
203 parameters	Δρ_min_ = −0.22 e Å^−^^3^
0 restraints	Extinction correction: *SHELXL97* (Sheldrick, 2008), Fc^*^=kFc[1+0.001xFc^2^λ^3^/sin(2θ)]^-1/4^
Primary atom site location: structure-invariant direct methods	Extinction coefficient: 0.0044 (4)

### Special details

**Table:**

Geometry. All e.s.d.'s (except the e.s.d. in the dihedral angle between two l.s. planes) are estimated using the full covariance matrix. The cell e.s.d.'s are taken into account individually in the estimation of e.s.d.'s in distances, angles and torsion angles; correlations between e.s.d.'s in cell parameters are only used when they are defined by crystal symmetry. An approximate (isotropic) treatment of cell e.s.d.'s is used for estimating e.s.d.'s involving l.s. planes.
Refinement. Refinement of *F*^2^ against ALL reflections. The weighted *R*-factor *wR* and goodness of fit *S* are based on *F*^2^, conventional *R*-factors *R* are based on *F*, with *F* set to zero for negative *F*^2^. The threshold expression of *F*^2^ > σ(*F*^2^) is used only for calculating *R*-factors(gt) *etc*. and is not relevant to the choice of reflections for refinement. *R*-factors based on *F*^2^ are statistically about twice as large as those based on *F*, and *R*-factors based on ALL data will be even larger.

### Fractional atomic coordinates and isotropic or equivalent isotropic displacement parameters (Å^2^)

**Table:**

	*x*	*y*	*z*	*U*_iso_*/*U*_eq_	
C1	0.11956 (11)	0.3349 (3)	0.21285 (9)	0.0330 (4)	
N1	0.09025 (10)	0.2129 (2)	0.16803 (8)	0.0384 (4)	
N2	0.16570 (9)	0.4615 (2)	0.19854 (7)	0.0356 (4)	
N3	0.10725 (10)	0.3212 (2)	0.26620 (7)	0.0384 (4)	
C2	0.12086 (14)	0.1763 (3)	0.11771 (10)	0.0493 (6)	
H2A	0.0856	0.2179	0.0784	0.074*	
H2B	0.1276	0.0534	0.1150	0.074*	
H2C	0.1697	0.2333	0.1264	0.074*	
C3	0.03122 (15)	0.0957 (3)	0.17362 (11)	0.0544 (6)	
H3A	0.0551	−0.0061	0.1964	0.082*	
H3B	−0.0021	0.0632	0.1323	0.082*	
H3C	0.0016	0.1525	0.1958	0.082*	
C4	0.24120 (12)	0.4984 (3)	0.24453 (11)	0.0467 (5)	
H4A	0.2483	0.4306	0.2818	0.070*	
H4B	0.2445	0.6194	0.2552	0.070*	
H4C	0.2805	0.4697	0.2269	0.070*	
C5	0.13871 (12)	0.5590 (3)	0.14236 (9)	0.0351 (4)	
C6	0.05972 (12)	0.5794 (3)	0.11112 (10)	0.0426 (5)	
H6	0.0246	0.5297	0.1277	0.051*	
C7	0.03365 (14)	0.6734 (3)	0.05569 (10)	0.0506 (6)	
H7	−0.0191	0.6847	0.0349	0.061*	
C8	0.08444 (15)	0.7502 (3)	0.03083 (10)	0.0529 (6)	
H8	0.0665	0.8125	−0.0067	0.063*	
C9	0.16233 (15)	0.7334 (3)	0.06238 (11)	0.0529 (6)	
H9	0.1971	0.7867	0.0462	0.064*	
C10	0.18983 (13)	0.6391 (3)	0.11746 (10)	0.0451 (5)	
H10	0.2426	0.6291	0.1380	0.054*	
C11	0.11811 (11)	0.4595 (3)	0.30767 (9)	0.0343 (4)	
C12	0.14071 (12)	0.4207 (3)	0.37217 (9)	0.0381 (5)	
H12	0.1473	0.3054	0.3849	0.046*	
C13	0.15342 (12)	0.5484 (3)	0.41700 (9)	0.0389 (5)	
H13	0.1689	0.5200	0.4596	0.047*	
C14	0.14288 (11)	0.7195 (3)	0.39797 (9)	0.0359 (5)	
C15	0.11713 (12)	0.7640 (3)	0.33462 (9)	0.0400 (5)	
H15	0.1088	0.8795	0.3224	0.048*	
C16	0.10416 (12)	0.6349 (3)	0.29011 (9)	0.0403 (5)	
H16	0.0858	0.6639	0.2476	0.048*	
N4	0.15796 (10)	0.8552 (2)	0.44528 (8)	0.0420 (4)	
O1	0.17013 (11)	0.8141 (2)	0.50039 (7)	0.0594 (5)	
O2	0.15764 (12)	1.0061 (2)	0.42853 (9)	0.0682 (6)	

### Atomic displacement parameters (Å^2^)

**Table:**

	*U*^11^	*U*^22^	*U*^33^	*U*^12^	*U*^13^	*U*^23^
C1	0.0320 (10)	0.0350 (10)	0.0298 (9)	0.0008 (8)	0.0075 (8)	0.0000 (8)
N1	0.0432 (10)	0.0390 (9)	0.0322 (8)	−0.0066 (8)	0.0116 (7)	−0.0069 (7)
N2	0.0323 (8)	0.0417 (10)	0.0296 (8)	−0.0062 (7)	0.0062 (7)	−0.0008 (7)
N3	0.0466 (10)	0.0382 (10)	0.0318 (8)	−0.0040 (8)	0.0148 (7)	−0.0027 (7)
C2	0.0575 (14)	0.0557 (14)	0.0345 (11)	0.0041 (12)	0.0152 (10)	−0.0095 (10)
C3	0.0602 (15)	0.0503 (14)	0.0496 (13)	−0.0181 (12)	0.0141 (11)	−0.0088 (11)
C4	0.0341 (11)	0.0515 (14)	0.0465 (12)	−0.0057 (10)	0.0027 (9)	0.0047 (10)
C5	0.0376 (10)	0.0360 (11)	0.0314 (9)	−0.0010 (9)	0.0113 (8)	−0.0025 (8)
C6	0.0363 (11)	0.0529 (13)	0.0386 (11)	−0.0004 (10)	0.0124 (9)	0.0043 (10)
C7	0.0445 (13)	0.0609 (15)	0.0408 (12)	0.0045 (11)	0.0068 (10)	0.0038 (11)
C8	0.0671 (16)	0.0527 (14)	0.0349 (11)	−0.0012 (12)	0.0118 (11)	0.0083 (11)
C9	0.0603 (15)	0.0551 (15)	0.0477 (13)	−0.0103 (12)	0.0237 (12)	0.0064 (11)
C10	0.0412 (12)	0.0511 (13)	0.0430 (12)	−0.0064 (10)	0.0142 (9)	0.0027 (10)
C11	0.0327 (10)	0.0400 (11)	0.0318 (10)	−0.0023 (8)	0.0127 (8)	−0.0015 (9)
C12	0.0458 (12)	0.0339 (11)	0.0343 (10)	0.0012 (9)	0.0128 (9)	0.0033 (9)
C13	0.0430 (11)	0.0448 (12)	0.0290 (10)	0.0014 (10)	0.0118 (8)	0.0033 (9)
C14	0.0364 (11)	0.0397 (11)	0.0335 (10)	0.0008 (9)	0.0141 (8)	−0.0054 (9)
C15	0.0475 (12)	0.0356 (11)	0.0388 (11)	0.0094 (9)	0.0167 (9)	0.0031 (9)
C16	0.0471 (12)	0.0445 (12)	0.0279 (10)	0.0078 (10)	0.0108 (9)	0.0036 (9)
N4	0.0435 (10)	0.0426 (11)	0.0429 (10)	−0.0015 (8)	0.0184 (8)	−0.0078 (8)
O1	0.0782 (12)	0.0658 (12)	0.0362 (8)	−0.0074 (9)	0.0215 (8)	−0.0125 (8)
O2	0.1042 (16)	0.0391 (10)	0.0635 (11)	−0.0023 (10)	0.0309 (11)	−0.0080 (8)

### Geometric parameters (Å, º)

**Table:**

C1—N3	1.298 (2)	C7—C8	1.374 (3)
C1—N1	1.353 (2)	C7—H7	0.9300
C1—N2	1.401 (3)	C8—C9	1.377 (3)
N1—C2	1.451 (3)	C8—H8	0.9300
N1—C3	1.451 (3)	C9—C10	1.379 (3)
N2—C5	1.411 (2)	C9—H9	0.9300
N2—C4	1.457 (2)	C10—H10	0.9300
N3—C11	1.387 (2)	C11—C12	1.402 (3)
C2—H2A	0.9600	C11—C16	1.408 (3)
C2—H2B	0.9600	C12—C13	1.372 (3)
C2—H2C	0.9600	C12—H12	0.9300
C3—H3A	0.9600	C13—C14	1.381 (3)
C3—H3B	0.9600	C13—H13	0.9300
C3—H3C	0.9600	C14—C15	1.386 (3)
C4—H4A	0.9600	C14—N4	1.452 (3)
C4—H4B	0.9600	C15—C16	1.375 (3)
C4—H4C	0.9600	C15—H15	0.9300
C5—C10	1.390 (3)	C16—H16	0.9300
C5—C6	1.397 (3)	N4—O2	1.223 (2)
C6—C7	1.382 (3)	N4—O1	1.226 (2)
C6—H6	0.9300		
			
N3—C1—N1	118.90 (18)	C8—C7—C6	121.0 (2)
N3—C1—N2	125.16 (18)	C8—C7—H7	119.5
N1—C1—N2	115.81 (16)	C6—C7—H7	119.5
C1—N1—C2	123.70 (18)	C7—C8—C9	118.8 (2)
C1—N1—C3	119.52 (17)	C7—C8—H8	120.6
C2—N1—C3	116.34 (18)	C9—C8—H8	120.6
C1—N2—C5	121.22 (16)	C8—C9—C10	121.3 (2)
C1—N2—C4	118.76 (16)	C8—C9—H9	119.4
C5—N2—C4	119.93 (17)	C10—C9—H9	119.4
C1—N3—C11	121.92 (18)	C9—C10—C5	120.1 (2)
N1—C2—H2A	109.5	C9—C10—H10	119.9
N1—C2—H2B	109.5	C5—C10—H10	119.9
H2A—C2—H2B	109.5	N3—C11—C12	117.26 (18)
N1—C2—H2C	109.5	N3—C11—C16	125.34 (17)
H2A—C2—H2C	109.5	C12—C11—C16	117.30 (18)
H2B—C2—H2C	109.5	C13—C12—C11	121.7 (2)
N1—C3—H3A	109.5	C13—C12—H12	119.1
N1—C3—H3B	109.5	C11—C12—H12	119.1
H3A—C3—H3B	109.5	C12—C13—C14	119.09 (18)
N1—C3—H3C	109.5	C12—C13—H13	120.5
H3A—C3—H3C	109.5	C14—C13—H13	120.5
H3B—C3—H3C	109.5	C13—C14—C15	121.30 (19)
N2—C4—H4A	109.5	C13—C14—N4	119.30 (18)
N2—C4—H4B	109.5	C15—C14—N4	119.39 (19)
H4A—C4—H4B	109.5	C16—C15—C14	119.0 (2)
N2—C4—H4C	109.5	C16—C15—H15	120.5
H4A—C4—H4C	109.5	C14—C15—H15	120.5
H4B—C4—H4C	109.5	C15—C16—C11	121.35 (18)
C10—C5—C6	118.56 (19)	C15—C16—H16	119.3
C10—C5—N2	120.95 (19)	C11—C16—H16	119.3
C6—C5—N2	120.48 (18)	O2—N4—O1	122.59 (19)
C7—C6—C5	120.2 (2)	O2—N4—C14	118.72 (18)
C7—C6—H6	119.9	O1—N4—C14	118.70 (19)
C5—C6—H6	119.9		
			
N3—C1—N1—C2	−157.1 (2)	C8—C9—C10—C5	−0.2 (4)
N2—C1—N1—C2	18.9 (3)	C6—C5—C10—C9	−1.3 (3)
N3—C1—N1—C3	15.0 (3)	N2—C5—C10—C9	179.8 (2)
N2—C1—N1—C3	−169.05 (19)	C1—N3—C11—C12	−149.4 (2)
N3—C1—N2—C5	−130.6 (2)	C1—N3—C11—C16	34.4 (3)
N1—C1—N2—C5	53.7 (3)	N3—C11—C12—C13	179.57 (19)
N3—C1—N2—C4	46.0 (3)	C16—C11—C12—C13	−3.9 (3)
N1—C1—N2—C4	−129.7 (2)	C11—C12—C13—C14	0.7 (3)
N1—C1—N3—C11	−163.60 (18)	C12—C13—C14—C15	2.3 (3)
N2—C1—N3—C11	20.8 (3)	C12—C13—C14—N4	−178.60 (18)
C1—N2—C5—C10	−158.0 (2)	C13—C14—C15—C16	−2.0 (3)
C4—N2—C5—C10	25.4 (3)	N4—C14—C15—C16	178.97 (19)
C1—N2—C5—C6	23.1 (3)	C14—C15—C16—C11	−1.4 (3)
C4—N2—C5—C6	−153.4 (2)	N3—C11—C16—C15	−179.51 (19)
C10—C5—C6—C7	1.9 (3)	C12—C11—C16—C15	4.2 (3)
N2—C5—C6—C7	−179.2 (2)	C13—C14—N4—O2	171.2 (2)
C5—C6—C7—C8	−1.0 (4)	C15—C14—N4—O2	−9.8 (3)
C6—C7—C8—C9	−0.5 (4)	C13—C14—N4—O1	−9.2 (3)
C7—C8—C9—C10	1.1 (4)	C15—C14—N4—O1	169.88 (19)

### Hydrogen-bond geometry (Å, º)

**Table:**

*D*—H···*A*	*D*—H	H···*A*	*D*···*A*	*D*—H···*A*
C12—H12···O2^i^	0.93	2.49	3.416 (3)	173
C2—H2*A*···O1^ii^	0.96	2.72	3.064 (3)	102

Symmetry codes: (i) *x*, *y*−1, *z*; (ii) *x*, −*y*+1, *z*−1/2.
